# The free moment in walking and its change with foot rotation angle

**DOI:** 10.1186/1758-2555-1-19

**Published:** 2009-08-13

**Authors:** Sivan Almosnino, Tara Kajaks, Patrick A Costigan

**Affiliations:** 1 Biomechanics and Ergonomics Laboratory, School of Kinesiology and Health Studies, 69 Union St., Queen's University, Kingston, ON, K7L 3N6, Canada; 2 Human Mobility Research Centre, Kingston General Hospital, 76 Stewart St., Kingston, ON, K7L 2V7, Canada; 3 Biomechanics Laboratory, Department of Kinesiology, McMaster University, 1280 Main St. West, Hamilton, ON, L8S 4K1, Canada

## Abstract

**Background:**

This investigation characterized the time-history pattern of the free moment (FM) during walking and, additionally, assessed whether walking with either an internally or externally rotated foot position altered the FM's time-history.

**Methods:**

Force plate and foot kinematic data were acquired simultaneously for 11 healthy subjects (6 males, 5 females) while walking at their self-selected comfortable speed in 3 foot rotation conditions (normal, internal and external). The FM was calculated and normalized by the product of each participant's body weight and height prior to extraction of peak FM, occurrence of peak FM in stance and net relative impulse. Differences in these values across foot rotation conditions were assessed using separate one-way, repeated measures analysis of variance and subsequent pair-wise comparisons.

**Results:**

The average FM pattern during normal walking exhibits a biphasic shape: resisting inward rotation during approximately the first half of stance and outward rotation during the latter part of stance. While no differences in peak FM or net relative impulse were observed between the internal foot rotation condition and normal walking, the external foot rotation condition resulted in significantly greater peak FM and relative net impulse in comparison to normal walking.

**Conclusion:**

The differences in selected FM variables between normal walking and the external foot rotation condition are attributable to individual subject response to walking with an externally rotated foot. In this condition, some subjects displayed a FM pattern that was similar to that recorded during normal walking, while others displayed markedly larger FM patterns that are comparable in magnitude to those reported for running. The larger FM values in these latter subjects are speculated to be a result of excessive transverse plane body movements. Whilst further investigation is warranted regarding the FM time-history characteristics during walking, our results indicate that the FM may provide useful information in assessment of gait.

## Background

**Figure 1:**
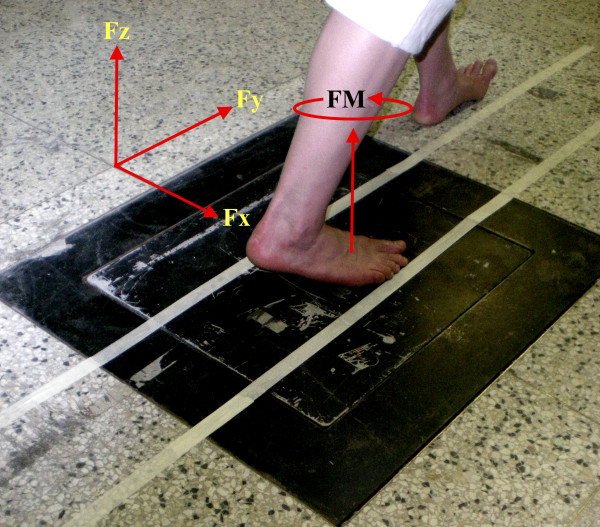
**Experimental procedures**. White parallel lines placed over the walkway aided subjects in reproducing the foot rotation angle in the internal (INT) and external (EXT) conditions. The FM is depicted as acting vertically through the hypothetical location of the center of pressure at this time instant, and its direction is in accordance with the force plate coordinate system used in this investigation.

Ground reaction force (GRF) time-histories, measured predominantly using floor embedded force plates, have been documented extensively in both normal and pathological populations and for a variety of human ambulatory activities (e.g. [1-6]). The majority of investigations that quantify GRF patterns focus on forces acting along the primary, orthogonal axes (i.e. vertical, anterior-posterior and medio-lateral). An additional force plate measure rarely reported is the free moment (FM) (table 1). The FM is the reaction to the force couple exerted by the foot on the ground acting about a vertical axis originating at the foot's center of pressure (*CoP*) (figure 1) [7,8].

**Table 1:** Investigations reporting upon the free moment (FM) during running and walking.

**Author(s), year**	**Activity**	**Subjects**	**FM** **Normalization**	**Peak FM (± SD) †**
Nigg et al, 1982 [34] ‡	Walking (w) & Running (r)	16, unilateral ACL insufficiency	None	(w) Injured limb 12.9 Nm (w) Normal limb 11.5 Nm (r) Injured Limb 15.9 Nm (r) Normal Limb 17.5 Nm

Nigg, 1986 [7] §	Running (3.5 ms^-1)^	1, rear foot striker.	None	Range across different footwear conditions5-7 Nm

Holden & Cavanagh, 1991 [8] £	Running (4.5 ± 10% ms^-1^)	10, male, 'normal foot arches'.	BW × ht	'Varus' shoes 6.7 ± 1.6 × 10^-3^ 'Neutral' shoes 9.7 ± 1.6 × 10^-3^ 'Valgus' shoes 12.4 ± 1.6 × 10^-3^

Milner et al, 2006 [9]	Running (3.7 ± 5% ms^-1^)	***Control Group***: 25, mixed, healthy. ***Experimental Group***: 25, female, history of tibial stress fractures.	BW × ht	5.9 ± 2.1 × 10^-3^ 9.3 ± 4.3 × 10^-3^

Creaby & Dixon, 2008 [33]	Running (3.6 ± 5% ms^-1^)	***Control Group***: 20 military recruits, no lower extremity injuries. ***Experimental Group***: 10 military recruits, sustained tibial stress fracture.	BW × ht	9.3 ± 3.2 × 10^-3^ 9.5 ± 2.1 × 10^-3^

Li et al, 2001 [13] §	Walking ('low', 'comfortable', 'fast')	17 total (5 adult males, 6 adult females, 6 children).	BW	Range across speed conditions, adults only 2.5 - 10.0 × 10^-3^

Umberger, 2008 [14] §	Walking (1.3 ms^-1^)	5 male, 3 female, healthy.	BW × LL	Range 0.5-1.5 × 10^-2^

BW = bodyweight, ht = height, LL = leg length

† Note differences in peak FM magnitude between studies due to different normalization procedures.

‡ Nigg et al (1982) do not provide ambulation speeds, participant sex or peak FM dispersions measures.

£ Holden and Cavanagh (1991) report peak FM variability using the standard error of the mean (SEM).

Values were converted to SD by multiplying the SEM by the square root of the sample size (n = 10).

§ Peak FM values not explicitly given. Approximate values are listed based on visual estimation of depicted FM time histories.

Recently, attention has been drawn to the clinical usefulness of the FM by Milner et al [9], who were able to discriminate retrospectively, based on FM indices, between a group of healthy runners and a group of female runners with a history of tibial stress fractures. Based on the results of Milner et al [9], it can be speculated that the FM might serve as an indicator for the amount of torsional loading experienced during ambulation. While this supposition has yet to be validated, there are two related points that make it appealing: one, cortical bone is not able to sustain large amounts of torsional loading [10,11], and, two, considerable torsional loading is experienced during ambulation, specifically during the push off phase of walking [12]. The question that naturally arises is whether an elevated FM during walking a potential cause of tibial stress fractures?

An elevated FM may be due to individual gait characteristics. Specifically, the FM has been found to be sensitive to gait modifications employed in the transverse plane: both Li et al [13] and Umberger [14] observed temporal and amplitude dissimilarities in the FM pattern when subjects walked across a force platform with and without arm swing, a movement that exerts a transverse force couple on the ground during walking. The vertical and anterior-posterior GRF components, on the other hand, were virtually indistinguishable across the two arm conditions [14].

Another gait modification employed in the transverse plane, and that might influence the FM, is the foot rotation angle adopted during walking. Modification of the foot rotation angle during walking as been documented to occur naturally as a function of the goals of the ambulatory task, or may be artificially induced as part of an interventional program. For example, it has been suggested that the adaptation of an internally rotated foot position during walking minimizes the resistive moment that must be overcome by the ankle plantar flexors during the push-off phase of stance [15]. This is achieved by effectively reducing the moment arm of the GRF vector with respect to the talocrural joint axis. Eredmir and Piazza [15] note that modifying the foot rotation angle to a more internal one has been observed in high school aged subjects during sprint-running [16], and in subjects walking while carrying external loads [17]. In contrast, several investigations have demonstrated that adapting a more externally rotated foot position decreases the magnitude of the knee adduction moment, thus unloading the medial compartment of the knee [18-21]. This is achieved by shortening the moment arm of the GRF in relation to the knee joint center in the frontal plane, primarily during the second half of stance [20]. While the influence of foot rotation on frontal knee and ankle moments has been examined, its influence on the transverse plane moments and the FM during walking has not been explored in adults.

While the influence of the foot rotation angle adopted during stance on the FM pattern is unclear, it can be postulated that, irrespective of the foot rotation position adopted relative to normal walking, attempting to align the foot with the direction of forward progress during late stance would require individuals to exert a twisting action of the foot on the ground that would subsequently alter the magnitude of the FM. Thus, the aim of this investigation is to test whether different foot rotation positions produce changes in the pattern of the FM pattern during walking. As part of this investigation, the pattern of the FM during normal walking will be described, consequently addressing the paucity of information related to this force plate measure.

## Methods

This investigation used an existing data set [22], for which the data collection procedures have been outlined [23-25]. These will be briefly described, as well as additional information pertaining to the aims of the current investigation.

### Subjects

A convenience sample of six males (age (mean ± SD) 23.9 ± 1.8 years, height 1.84 ± 0.07 meters, weight 819 ± 67 N) and five females (age 21.9 ± 0.8 years, height 1.67 ± 0.07 meters, weight 579 ± 63 N) volunteered to participate in the study. None of the subjects were suffering from or reported a history of lower extremity injury. Prior to testing, all subjects were briefed on the procedures of the investigation and subsequently gave their informed consent. Approval for this study was obtained from the Queen's University's Research Ethics Board.

### Equipment

Foot rotation angle during the stance phase, defined as the angle between the foot's long axis and the direction of forward progress, was calculated from the position of three active infrared-emitting diodes (IREDS) affixed directly to each subjects' right lateral malleoulus, tuber calcanei and 5^th ^metatarsal head. The IREDS were tracked in three dimensional space using two Optotrak position sensors (Northern Digital Inc., Waterloo, ON, Canada). Ground reaction force measurements were obtained using a strain-gauge force platform (model OR6-7-1000, AMTI Inc., Watertown, MA, USA). Both IRED and force plate data acquisition were sampled synchronously at 100 Hz. A calibration procedure was employed prior to data collection to align the coordinate systems of the force plate and Optotrak system.

### Procedures

Subsequent to marker placement, a static-standing calibration trial was performed with the subjects standing such that the middle-posterior aspect of the heel and 2^nd ^toe were aligned with the anterior-posterior force plate axis. All subsequent foot rotation angles are referenced to this position in the global coordinate system. Subjects were then asked to perform several practice trials while walking across the force platform at a self-selected comfortable speed. During these practice trials the normal (NORM) foot rotation angle of each of the subjects' right foot was determined. During testing, the subjects walked across the force platform normally (NORM) or while rotating their foot externally (EXT) or internally (INT) by approximately 30 degrees with respect to their NORM angle. To aid the subjects in reproducing the EXT and INT foot rotation angles during testing, two parallel lines were placed on the walkway (Figure 1). The gap between the two lines corresponded to each participant's individual foot length such that, when subjects walked with their foot filling the gap, the same foot rotation angle could be achieved. Subjects were given as many practice trials as needed in order to ensure their ability to walk comfortably while performing the INT and EXT conditions. The subjects performed five trials in each foot rotation condition (NORM, INT, EXT) in a random order. Arm swing was standardized across subjects and conditions by asking subjects to walk with their elbows bent at a 90 degree angle.

### Data Analysis

Prior to the extraction of the variables of interest, kinematic data were filtered using a 2^nd ^order, zero phase shift, low pass Butterworth filter with a cut-off frequency of 6 Hz. Calculation of the free moment was in accordance with a reaction-oriented, orthogonal force plate coordinate system, where the anterior-posterior axis (*Y*) points in the direction of forward progression, the vertical axis (*Z*) points upwards and the medio-lateral (*X*) axis points to the right (Figure 1). Thus, with respect to the right foot, a positive free moment opposes outward rotation. Conversely, a negative free moment opposes inward rotation. The calculation of the FM requires the force (*Fx, Fy, Fz*) and moment (*Mx, My, Mz*) components, as well as the location of the CoP, which was calculated as follows:

(1)

(2)

Where *CoP*_*x *_and *CoP*_*y *_are the positions of the center of pressure along the medio-lateral and anterior-posterior force plate axes, respectively, and *Z*_*off *_is the vertical distance offset between the surface and true center of the force plate. To control for erroneous *CoP *values at the beginning and end of stance due to division by small vertical forces (*Fz*), the calculation of the *CoP *was initiated and terminated when the *Fz *value was above 5% of the maximal value recorded during each trial. The FM is given by [26-28]:

(3)

Note that the *CoP *and FM calculations are only valid for the model of force plate used in this investigation.

All FM waveforms were amplitude-normalized to the product of each individual's body weight (N) and height (m). Additionally, the period of foot contact with the force plate was normalized to a uniform length of 101 data points, which represented 0 - 100% of the stance phase. To assess differences in FM between foot rotation conditions, the following dependent variables were extracted from each of the five trials performed in each condition and subsequently averaged per subject: peak free moment (PFM), occurrence of peak free moment in stance (OPFM) and the relative net impulse (IMP), which is the net area under the FM - stance curve [29]. We also report the un-normalized peak FM magnitudes for comparison with studies that have chosen not to normalize the FM.

### Statistical Analysis

Temporal similarity between pairs of FM waveforms (NORM vs. EXT and NORM vs. INT, respectively) was assessed using Pearson product moment correlation coefficient (*r*) [30,31], while differences in magnitude were assessed by the root mean square difference (RMSD). RMSD values were calculated separately for each subject and subsequently averaged across subjects [14]. With regards to the calculation of *r*, since correlation coefficients are not normally distributed, a Fisher Z transformation was applied to all individual *r *values, which were then averaged across subjects. Thereafter, the hyperbolic tangent of the average Z score was taken to obtain the average correlation coefficient [31].

Dependent measures were tested for differences between the three foot rotation conditions using a one-way, repeated measures analysis of variance (ANOVA) (p < 0.05). For all omnibus test comparisons, the degrees of freedom used to calculate *p *values were corrected using Greenhouse-Geisser sphericity estimates. Subsequently, pair-wise comparisons were used to assess differences between the NORM foot rotation condition and the INT and EXT foot rotation conditions, respectively. The alpha level for these comparisons was adjusted using a Sidak correction procedure, based on an *a priori *alpha level set at 0.05. In addition, estimates of effect size (ES) were calculated following the guidelines of Dunlop et al [32].

The relationship between the foot rotation angle adopted during stance and PFM and IMP was also assessed using Pearson product moment correlation coefficient (*r*). This was done in two ways: first, *r *was calculated between the 'absolute' foot rotation angle in each condition and the corresponding PFM and IMP values obtained. Second, *r *was calculated between the relative change in foot rotation angle in the INT and EXT foot rotation conditions and the corresponding change in PFM and IMP with respect to normal walking. This was done by subtracting the values recorded for each subject during the NORM walking condition from those obtained in the INT and EXT conditions.

Finally, differences in average gait speed and foot rotation angle in each of the three foot rotation conditions were assessed using a one way, repeated measures ANOVA and planned contrasts. The alpha level for these comparisons (*α *= 0.05) was adjusted using a Bonferonni correction procedure.

## Results

**Figure 2:**
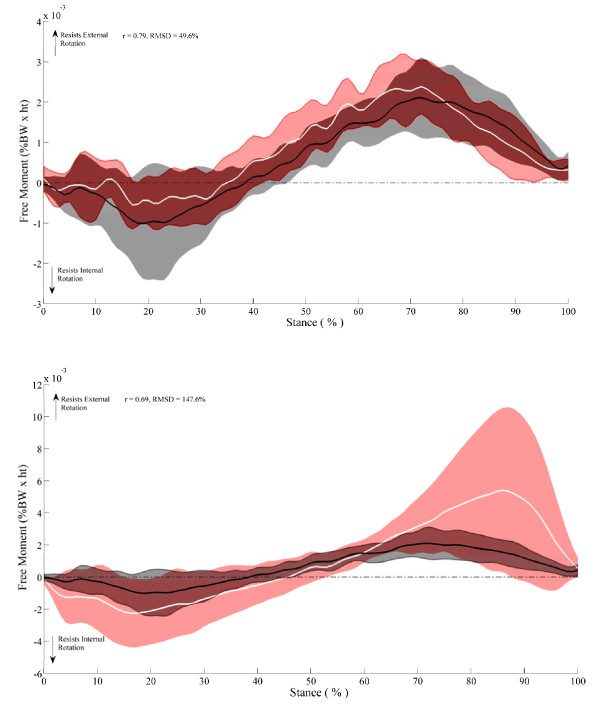
**The free moment time history pattern normal walking**. **a) **FM during normal walking (black line is average, grey shadows are ± 1 SD variability bands) and when walking with an internally rotated foot (white line is mean, red shadows are ± 1 SD variability bands) and **b) **FM during normal walking (black line is average, grey shadows are ± 1 SD variability bands) and when walking with an externally rotated foot (white line is average, red shadows are ± 1 SD variability bands).

**Figure 3:**
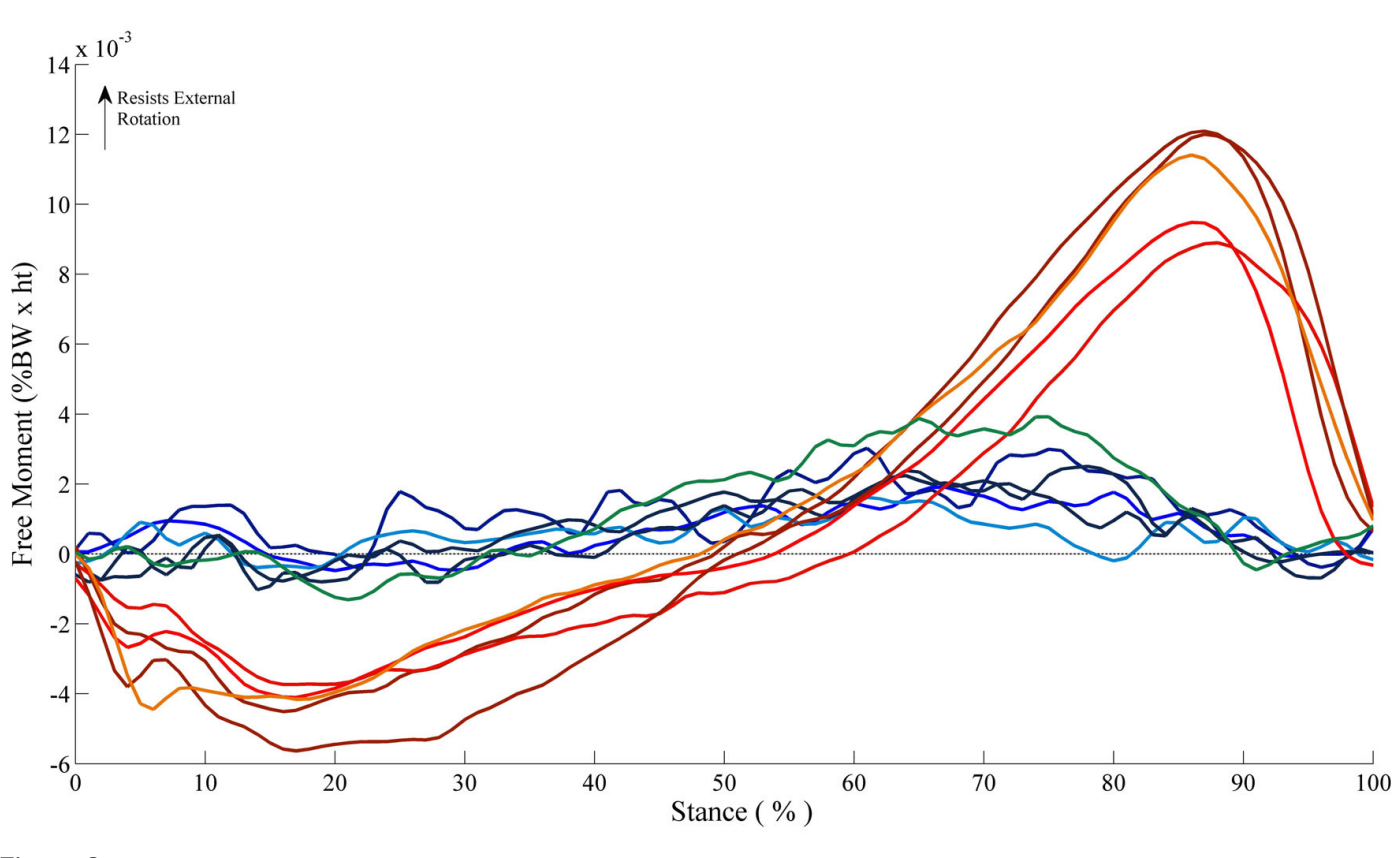
**Individual subject free moment time history pattern during walking with an externally (EXT) rotated foot**. Each line represents individual subject ensemble average curve of 5 trials in the external foot rotation walking condition.

Figure 2(a) and 2(b) depict the FM during normal walking and in the internal and external foot rotation conditions, respectively. In general, the FM in all three foot rotation conditions exhibits a biphasic shape whereby the FM initially resists inward rotation, then reverses just prior to mid stance to produce a positive FM that is indicative of resistance to outward rotation. Large inter-subject variability was present in the EXT foot rotation condition. Figure 3 shows that subjects could be characterized by one of two FM patterns, one similar in magnitude to that observed in the normal condition and another where the pattern demonstrated large excursions in magnitude during early and late stance. Evaluation of *r *values calculated between pairs of FM curves indicate that on average, the FM patterns for the INT and NORM foot rotation conditions are more similar in magnitude and temporal characteristics (*r *= 0.79, RMSD = 49.9%) than the EXT and NORM foot rotation conditions (*r *= 0.69, RMSD = 147.6%).

Results of the ANOVAs performed on selected FM variables (table 2) reveal that PFM and IMP were significantly affected by foot rotation condition. Consequently, pair-wise comparisons show differences in the NORM and EXT average PFM values (2.8 ± 0.8 × 10^-3 ^vs. 6.7 ± 4.1 × 10^-3^, respectively, p < 0.05, ES = 0.88) and in the IMP values (5.8 ± 3.2 × 10^-2 ^vs. 10.1 ± 4.3 × 10^-2^, respectively, p < 0.05, ES = 0.92). Conversely, no differences were found between the NORM and INT conditions in (p > 0.05 for both PFM and IMP), nor was a significant main effect detected in OPFM across the three foot rotation conditions (INT 70 ± 9%, NORM 69 ± 3%, EXT 77 ± 9%, p > 0.05). Effects size values were relatively small for all of these latter comparisons (ES range = -0.14 - 0.39), except for differences between NORM vs. EXT OPFM, which exhibited a moderate ES of 0.51.

**Table 2:** Foot rotation angle, walking speed and FM measures in each of the three foot rotation conditions.

	**Foot rotation condition**		
	
**Variable**	**Internal^§^**	**Normal**	**External^£^**
Foot Rotation Angle (degrees)^†^	-9.1 ± 7.9	18.5 ± 8.15	40.2 ± 8.7
Walking Speed (ms^-1^)	1.08 ± 0.16	1.10 ± 0.12	1.12 ± 0.15
Un-normalized Peak FM (Nm)*	3.9 ± 1.0	3.4 ± 1.4	8.8 ± 6.4
PFM (dimensionless, × 10^-3^)	3.2 ± 0.9	2.8 ± 0.8	6.7 ± 4.1^‡^
OPFM (% stance)	69 ± 3	70 ± 9	77 ± 9
IMP (× 10^-2^)	7.5 ± 2.7	5.8 ± 3.2	11.0 ± 4.1^‡^

PFM = normalized peak free moment, OPFM = occurrence of peak free moment, IMP = relative net impulse. All values presented as mean ± 1 SD.

* Un-normalized peak FM presented for the sake of comparison with results of other investigations.

† All foot rotation angles significantly different than each other (p < 0.016)

‡ Significantly different than normal walking condition (p < 0.05)

§ Effect size values for Norm vs. INT: PFM = 0.31, OPFM = -0.14, IMP = 0.39.

£ Effect size values for Norm vs. Ext: PFM = 0.88, OPFM = 0.51, IMP = 0.92.

The magnitudes of the relationships between the absolute foot rotation position and the magnitude of PFM and IMP were *r *= 0.39 and *r *= 0.28, respectively. Evaluation of the corresponding coefficient of determination values (*r*^2^) for these relationships suggest that very little of the variation seen in PFM or IMP may be explained by the absolute foot rotation angle adopted during stance (*r*^2 ^= 0.15 and 0.08 respectively). When expressed relative to the values recorded for the normal walking condition, the magnitudes of the relationships of foot rotation position and PFM and IMP were *r *= 0.65 and 0.31, respectively. Once more, the portion of the variation in relative PFM and IMP explained by foot rotation position adopted relative to normal walking is comparatively small (*r*^2 ^= 0.42 and 0.09, respectively).

The average foot rotation angle adopted in each foot rotation position was found to be significantly different (INT -9.1 ± 7.9°, NORM 18.5 ± 8.15°, EXT 40.2 ± 8.7°, p < 0.016 for all planned contrasts), with all subjects walking during individual trials within 2.8 degrees of their average foot rotation angle in each of the conditions. Finally, no significant main effect was detected for average walking speeds (INT 1.08 ± 0.16 ms^-1^, NORM 1.10 ± 0.12 ms^-1^, EXT 1.12 ± 0.15 ms^-1^, p > 0.05).

## Discussion

The primary aim of this investigation was to assess whether time-history differences exist in the FM when modifying the foot rotation angle during walking. In addition, a description of the characteristics of the FM during normal walking was presented. With regards to differences in FM patterns across the three foot rotation condition, we assumed that during late stance, alignment of the foot with the direction of forward progress would necessitate a twisting action of the foot on the ground that would alter the FM pattern. Based on this, it was expected that internal and external foot rotation condition would alter the FM pattern in different directions. Specifically, for the internally rotated foot position, we contemplated that abduction of the foot would occur during late stance and subsequently produce a positive FM. Conversely, in the externally rotated foot condition, we expected the foot to adduct during late stance and subsequently produce a negative FM pattern. In the latter condition the FM time-history is clearly altered, but not in the expected direction. There are several plausible explanations to the discrepancy between out hypothesis and the results obtained. The first explanation is that we did not consider that the FM reflects the sum of the force couples effects about a vertical axis [28]. As such, while the foot might be in fact attempting to align with the direction of forward progress during late stance, and consequently producing a negative FM, the movements of other body segments acting to generate a larger coupling effect in the opposite direction ultimately produce a net FM that is not reflective of the movement of the foot. Another explanation relates to the argument presented by Li et al [13] as to the role of the FM during the double support phase of stance, where the FM produced by both feet acts in the same direction to counteract the moment produced by the horizontal forces about the body's vertical axis. Thus, a reversal of the FM pattern during late stance would likely create an imbalance about the body's vertical axis, which would be unbeneficial as this may interfere with the goal of forward progression [13].

Additionally, changes in the FM pattern during late stance were evident in the externally rotated foot condition. In this condition, several participants displayed normalized peak FM values that are as large as those reported for running in either healthy participants or those who have suffered from tibial stress fractures [9,33]. The un-normalized peak FM values for these participants were as large as the average peak FM values reported by Nigg et al [34] for participants suffering from unilateral anterior cruciate ligament deficiency performing walking and running trials. Given that we standardized arm swing within subject and across conditions, the greater PFM values observed for some participants in the EXT condition suggests the existence of transverse plane movement modifications employed by other body segments. Unfortunately, we cannot identify what underlying movement modifications were made by those participants who exhibited greater FM values. However, it is interesting to note that changes in either absolute or relative PFM and IMP were not particularly dependent on the absolute or relative foot rotation angle adopted during stance. This may suggest that the greater FM values observed for some participants in the EXT condition may be related individual anatomical structural constraints, such as increased hip tightness, or perhaps due to asynchrony in subtalar and tibio-femoral rotations as a result of prolonged foot pronation [35].

We have mentioned in the introduction section that the FM may perhaps be reflective of the torsional loading experienced by the lower extremities during ambulation. However, this supposition may not hold true in light of the results presented and current knowledge on this topic. Specifically, Carter [12] found that torsional stresses during the push-off phase of walking are substantially greater than those recorded during running. If the FM is a proxy for torsional stresses than the PFM during the push-off phase in walking should be greater than that observed for running. Our results suggest that the PFM in walking is less than or equal to that for running, but not substantially greater. However, given the sensitivity of the FM to subject specific responses when asked to walk with an externally rotated foot, and inferring from the discussion of Milner et al [9], we contemplate that the FM may be indicative of individual gait characteristics that may predispose individuals to tibial stress fractures or other lower extremity injuries in which excessive transverse plane rotations are suspected to be part of the mechanism of injury [e.g. anterior knee pain]. As such, researchers in these topic areas might benefit from extracting the FM for purposes of differentiating between injured and non-injured participants, especially in prospective-type studies planning to utilize force plate data. In addition, the FM might be used for the evaluation of proposed interventional programs exploiting artificially-induced external foot rotation [e.g. [19,20]].

There are some limitations to the present study. The first is that the changes in FM pattern and magnitude seen in subjects in the EXT condition may be primarily a result of foot placement targeting of the force plate. This has been previously found to have an insignificant effect on the magnitude and variability of most time domain orthogonal GRF parameters [36,37], and since the FM is a function of these parameters we would expect similar results for the FM. The second limitation relates to the restriction of arm swing during walking. The standardization of arm swing was pertinent for comparisons across foot rotation conditions, as arm swing has been documented to affect the magnitude of the FM [13,14]. Whilst taking this into account, it should be noted that we employed an arm swing standardization procedure that is much less constraining then the ones employed by either Li et al [13] or Umberger [14]. However, future investigations are needed to establish whether changes in arm swing accompany changes in foot rotation position during walking, and subsequently how this affects the FM pattern.

## Conclusion

This study presented a description of the time-history of the FM in normal walking and the effect of foot rotation upon it. On average, the free moment during walking tends to oppose inward rotation during early to just before mid stance at which time it reverses to oppose outward rotation. When walking with an internally rotated foot, selected FM indices were not statistically different than those recorded for normal walking. Conversely, when walking with an externally rotated foot, peak normalized free moment and impulse were significantly greater than normal walking.

This study is one of less than a handful of investigations that has reported upon the FM during walking. Future research directions on the behaviour of the FM waveform in other walking conditions, and in different subject populations, would help facilitate our understanding of the FM as an objective gait assessment measure.

## Competing interests

The authors declare that they have no competing interests.

## Authors' contributions

SA conceived the study, and was responsible for data analysis, statistical analysis, and drafting of the manuscript. TA designed the experimental protocol, collected all data, and helped draft the manuscript. PAC was senior author, providing guidance and advice on all aspects of the study and, in addition, was responsible for the development of the software used for data analysis. All authors read and approved the final manuscript.
